# Bone Tissue Evaluation Indicates Abnormal Mineralization in Patients with Autoimmune Polyendocrine Syndrome Type I: Report on Three Cases

**DOI:** 10.1007/s00223-023-01077-0

**Published:** 2023-03-22

**Authors:** Saila Laakso, Tong Xiaoyu, Stéphane Blouin, Petra Keplinger, Ville-Valtteri Välimäki, Heikki Kröger, Outi Mäkitie, Markus A. Hartmann

**Affiliations:** 1 Children’s Hospital and Pediatric Research Center, University of Helsinki and Helsinki University Hospital, Stenbäckinkatu 9, Helsinki, Finland; 2 Research Program for Clinical and Molecular Metabolism, Faculty of Medicine, University of Helsinki, Helsinki, Finland; 3 Folkhälsan Research Center, Helsinki, Finland; 4 Department of Orthopedics, Kuopio Musculoskeletal Research Unit, University of Eastern Finland, and, Kuopio University Hospital, Kuopio, Finland; 5 Ludwig Boltzmann Institute of Osteology at Hanusch Hospital of OEGK and AUVA Trauma Centre Meidling, 1st Medical Department Hanusch Hospital, Vienna, Austria; 6 Vienna Bone and Growth Center, Vienna, Austria; 7 Department of Orthopedics, University of Helsinki and Helsinki University Hospital, Helsinki, Finland; 8 Department of Molecular Medicine and Surgery, Karolinska Institutet, and Clinical Genetics, Karolinska University Hospital, Stockholm, Sweden

**Keywords:** Autoimmune polyendocrinopathy-candidiasis-ectodermal dystrophy, Autoimmune regulator, Bone histomorphometry, Quantitative backscattered electron imaging

## Abstract

Autoimmune polyendocrine syndrome type-1 (APS1) is characterized by autoimmune manifestations affecting different organs from early childhood on. Immunological abnormalities, the resulting endocrinopathies, and their treatments may compromise bone health. For the first time in APS1, we analyzed transiliac bone biopsy samples by bone histomorphometry and quantitative backscattered electron imaging in three adult patients (female P1, 38 years; male P2, 47 years; male P3, 25 years). All had biallelic mutations in the autoimmune regulator gene and in addition to endocrinopathies, also significant bone fragility. Histomorphometry showed bone volume in the lower normal range for P1 (BV/TV, − 0.98 SD) and P3 (− 1.34 SD), mainly due to reduced trabecular thickness (TbTh, − 3.63 and − 2.87 SD). In P1, osteoid surface was low (OS/BS, − 0.96 SD); active osteoblasts and double labeling were seen only on cortical bone. P3 showed a largely increased bone turnover rate (BFR/BV, + 4.53 SD) and increased mineralization lag time (Mlt, + 3.40 SD). Increased osteoid surface (OS/BS, + 2.03 and + 4.71 SD for P2 and P3) together with a large proportion of lowly mineralized bone area (Trab CaLow, + 2.22 and + 9.81 SD for P2 and P3) and focal mineralization defects were consistent with abnormal mineralization. In all patients, the density and area of osteocyte lacunae in cortical and trabecular bone were similar to healthy adults. The bone tissue characteristics were variable and included decreased trabecular thickness, increased amount of osteoid, and abnormal mineralization which are likely to contribute to bone fragility in patients with APS1.

**Supplementary Information:**

The online version contains supplementary material available at 10.1007/s00223-023-01077-0.

## Introduction

Autoimmune polyendocrine syndrome type 1 (APS1, OMIM #240,300), also known as autoimmune polyendocrinopathy-candidiasis-ectodermal dystrophy is a rare autoimmune disease with mutations in the autoimmune regulator (*AIRE*) gene. These lead to impaired expression of tissue-specific proteins in thymus, resulting in failure of negative selection of self-reactive T cells [1] and impaired regulatory T cell function [2]. Defects translate into organ-specific autoimmune diseases [3]. Majority of patients develop hypoparathyroidism and/or adrenocortical insufficiency during childhood requiring lifelong treatment [3].

Immunological abnormalities, endocrinopathies, and their treatments may impact bone health in patients with APS1. Our previous study associated impaired bone characteristics with disease severity [4]. We found multiple non-spinal fractures in 23% and spinal compression fractures in 7% of patients. Peripheral quantitative computer tomography (pQCT) showed reduced cortical thickness and trabecular volumetric bone mineral density (BMD) in patients with ≥ 7 disease manifestations while in more mildly affected patients these values were similar to controls [4].

We therefore set out to further examine the bone properties in patients with APS1. Here we provide a histomorphometric, mineral content, and osteocyte lacunae analysis of bone tissue in transiliac bone biopsy samples of three APS1 patients with osteoporotic BMD or vertebral compression fractures.

## Patients and Methods

### Patients

During a cross-sectional study on APS1, we evaluated comprehensively bone health in 44 patients. Osteoporotic BMD was found in four adults, and vertebral fractures in three additional patients [4]. Four of these seven patients were eligible for a bone biopsy procedure for research purposes, and three of them gave the informed consent to participate in the study. This study followed the principles of the Declaration of Helsinki and was approved by the Helsinki University Hospital ethics committee (HUS/1088/2016).

### Clinical Studies

Data on disease manifestations, their treatments, and fracture history were reviewed. Biochemical parameters were evaluated on the day of biopsy from peripheral blood obtained in the morning after an overnight fast. BMD was measured with dual-energy x-ray assessment and bone characteristics with pQCT as described previously [4]. T-scores for the total volumetric BMD (vBMD) and trabecular vBMD at distal site as well as for cortical vBMD at proximal radial site were calculated according to references [5, 6].

### Bone Biopsies

Transiliac bone biopsy samples of the anterior superior iliac crest were obtained with a trephine having an inner diameter of 7.5 mm (Rochester Bone Biopsy; Medical Innovations International, Rochester, MN, USA). The preceding tetracycline labeling followed a routine protocol: two courses of oral tetracycline (500 mg twice daily for 2 consecutive days), with a 12-day interval, with biopsy obtained 5 days after the second course.

### Sample Preparation and Bone Histomorphometry

Samples were fixed in ethanol (70%), gradually dehydrated with increasing concentrations of ethanol and embedded in polymethylmethacrylate according to standard protocols[7]. 5-μm-thick sections were stained with modified Masson Goldner trichrome stain. Quantitative bone histomorphometry was performed on the complete cancellous bone area using Osteomeasure system (OsteoMetrics, Atlanta, GA, USA). The nomenclature and parameters follow the recommendations by the American Society for Bone and Mineral Research [8]. Each sample was evaluated under bright light, polarized light, and fluorescence microscopy using a magnification of 200-fold. Z-values for histomorphometric parameters were calculated according to Rehman et al. [9].

### Bone Mineralization Density Distribution (BMDD)

The procedure to obtain BMDD curves using quantitative backscattered electron imaging (qBEI) is described in detail elsewhere [10]. The BMDD curve is the frequency distribution of measured calcium content normalized to 100% bone area. The curve is characterized by 5 parameters: (i) CaMean, the mean calcium content, (ii) CaPeak, the most frequent calcium concentration, (iii) CaWidth, the full width at half maximum of the curve reflecting the mineralization heterogeneity, (iv) CaLow and (v) CaHigh measure the amount of bone area mineralized below the 5th and above the 95th percentile of the adult trabecular reference curve, respectively. BMDD curves were obtained separately for trabecular and cortical compartments and compared to healthy adult reference curves published by Hartmann et al. [10].

### Osteocyte Lacunae Sections

Osteocyte lacunae sections (OLS) were analyzed on images obtained from calibrated qBEI measurements with a resolution increased to 0.88 µm per pixel. The size of the OLS was determined by setting a calcium threshold of 5.2 wt%. Parameters calculated were OLS-density (the number of osteocyte lacunae per bone area) and OLS-area (the mean area of OLS). OLS were defined as pores between 5 and 200 µm^2^ in size—see detailed description in Mähr et al. [11]. Data were compared against results from two healthy adults published previously [12].

## Results

### Clinical Data

The clinical characteristics of the three adult patients with APS1 (female P1: 38 years; male P2: 47 years; male P3: 25 years) are shown in Table 1. As signs of osteoporosis, P1 had experienced a vertebral compression fracture, whereas P2 and P3 had osteoporotic BMD. P3 had experienced five fractures due to high-energy accidents; all the fractures had been treated successfully with immobilization 4–12 years before bone biopsy. None of the patients were treated with bisphosphonates or other osteoporosis medications prior to biopsy.

**Table 1:** Clinical characteristics of the three patients with APS1

Characteristic	Patient 1	Patient 2	Patient 3
Gender	Female	Male	Male
Age (year)	38	47	25
Height (cm/Z-score)	152/− 2.9	167/− 2.3	175/− 0.9
BMI (kg/m^2^)	22.2	20.9	16.2
S-Ca-ion (mmol/L)	1.11 (1.16–1.30)	1.15 (1.16–1.30)	1.19 (1.16–1.30)
P-Pi (mmol/L)	1.35 (0.71–1.53)	0.7 (0.71–1.53)	1.29 (0.71–1.53)
S-D-25-OH (nmol/L)	65 (> 50)	90 (> 50)	128 (> 50)
P-Cr (μmol/L)	82 (50–90)	97 (60–100)	78 (60–100)
Lumbar spine BMD T-score	+ 1.6	− 1.7	− 3.3
Whole body BMD T-score	+ 1.2	− 2.8	− 3.5
Femoral neck BMD T-score	+ 1.0	− 2.2	− 2.9
Radial total vBMD T-score	− 1.4	− 4.9	− 1.5
Radial trabecular vBMD T-score	− 0.3	− 1.3	+ 0.7
Radial cortical vBMD T-score	+ 1.3	− 1.8	+ 1.2
*AIRE* genotype	c.769C > T/c.769C > T	c.769C > T/c.769C > T	c.769C > T/c.967_979del13
Disease manifestations (duration in years)	Hypoparathyroidism (33), adrenal insufficiency (30), hypogonadism (21), hypothyroidism, autoimmune gastritis, hepatitis (inactive), vitiligo, alopecia, recurrent oral candidiasis, intestinal dysfunction, gallbladder stones	Adrenal insufficiency (40), hypoparathyroidism (5), recurrent oral candidiasis, iritis, alopecia, autoimmune gastritis, intestinal dysfunction	Hepatitis (inactive), vitiligo, hypogonadism (11), exocrine pancreas insufficiency, growth hormone deficiency, diabetes, renal tubular acidosis (14), bronchiolitis associated with bronchiectasias, keratitis, recurrent oral candidiasis, enamel dysplasia, nail dystrophy
Hydrocortisone dose (mg/kg/day)	0.29	0.34	Only in case of stress
Other medications	Calcium, alfacalcidol, fludrocortisone, thyroxin, magnesium, contraceptive pills, vitamin B12, trimetoprime sulfa, iron	Calcium, magnesium, alfacalcidol, potassium, vitamin B12	Potassium, sodium bicarbonate, magnesium, vitamin D3, Sustanon, immunoglobulin G, Creon, trimetoprime sulfa, escitalopram, insulin

*S* serum; *Ca-ion* ionized calcium; *P* plasma; *Pi* phosphate; *D-25-OH* 25-hydroxy vitamin D; *Cr* creatinine; *BMD* bone mineral density; *vBMD* volumetric bone mineral density; *AIRE* autoimmune regulator gene. In parenthesis are indicated the normal range

All patients had biallelic *AIRE* mutations and 7–10 disease manifestations. P1 and P2 had both hypoparathyroidism and adrenal insufficiency, while P3 did not have hypoparathyroidism and needed hydrocortisone substitution only in the case of stress for early adrenal insufficiency. P1 and P3 had hypergonadotropic hypogonadism treated with contraceptive pills containing ethinylestradiol and with testosterone, respectively. P3 had stable renal tubular acidosis treated adequately with sodium substitution and sodium bicarbonate. Exocrine pancreas insufficiency was treated with enzyme substitution at meals in P3. Due to intestinal dysfunction, P1 had used lactobacillus supplement for years and P2 avoided dairy products.

P1 and P2 had short stature, whereas P3 was under-weighted. P1 and P2 had ionized calcium level slightly below normal range, but within the target range for patients with hypoparathyroidism. Whole body BMD T-scores were + 1.2, − 2.8, and − 3.5 for P1, P2, and P3, respectively.

### Bone Histomorphometry

**Fig. 1:**
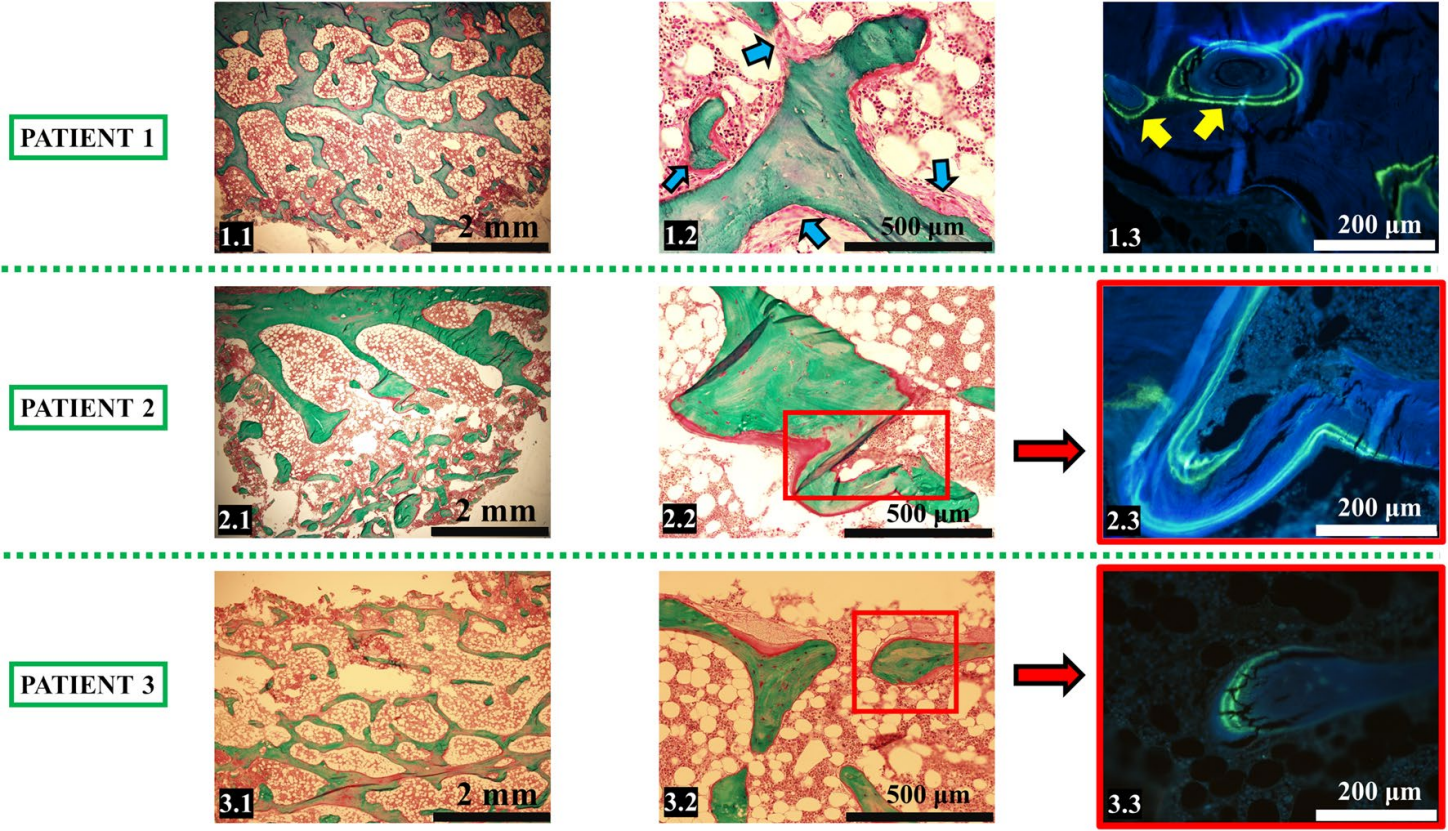
Typical microarchitectures of iliac crest specimens under light microscopy (1.1–1.2; 2.1–2.2; 3.1–3.2) and fluorescence microscopy (1.3; 2.3; 3.3) are exemplified from patients 1, 2 and 3 with APS1. The blue arrows indicated the peri-trabecular fibrosis and the double tetracycline labels seen in cortical bone were shown by yellow arrows in patient 1. The magnified images (highlighted by red rectangle) demonstrated the normal double labeling in cancellous bone of patient 2 and patient 3. Masson Goldner trichrome stain

In bone biopsies, bone volume (BV/TV) was in the normal range for all patients (Table 2, Fig. 1). Nevertheless, it was in the lower range for P1 and P3, which was mainly due to reduced trabecular thickness, but partly reversed by an increase in trabecular number.

**Table 2:** Bone histomorphometry results for the three patients with APS1. Z-scores in parenthesis are calculated according to Rehman et al.[9]

	Patient 1	Patient 2	Patient 3
BV/TV [%], bone volume/tissue volume	17.89 (− 0.98)	23.03 (+ 0.21)	17.28 (− 1.34)
Tb.Th [μm], trabecular thickness	**77.08 (**− **3.63)**	120.38 (− 0.62)	**63.45 (**− **2.87)**
Tb.N [1/mm], trabecular number	2.32 (+ 1.55)	1.91 (+ 0.53)	**2.72 (+ 2.55)**
OV/BV [%], osteoid volume	2.27 (− 0.83)	3.00 (− 0.08)	**7.90 (+ 2.26)**
OS/BS [%], osteoid surface	11.65 (− 0.96)	**27.50 (+ 2.03)**	**41.06 (+ 4.71)**
O.Th [μm], osteoid thickness	7.46 (− 0.41)	6.46 (− 0.75)	6.04 (− 0.8)
Ob.S/BS [%], osteoblast surface	x	4.41 (− 0.38)	2.26 (− 1.57)
ES/BS [%], eroded surface / bone surface	**7.73 (+ 3.03)**	4.48 (+ 0.21)	2.74 (− 0.80)
Oc.S/BS [%], osteoclast surface	0.61 (+ 0.56)	0.67 (+ 0.35)	x
MS/BS [%], mineralizing surface	x	8.85 (0.38)	8.29 (+ 0.26)
MAR [μm/day], mineral apposition rate	x	0.70 (+ 0.42)	0.69 (+ 0.42)
Aj.AR [μm/day], adjusted apposition rate	x	0.23 (− 0.35)	**0.14 (**− **2.20)**
BFR/BV [%/year], bone formation rate/bone volume (bone turnover rate)	x	37.64 (+ 0.73)	**65.68 (+ 4.53)**
Mlt [days], mineralization lag time	x	28.62 (+ 1.66)	**43.41 (+ 3.40)**

Deviations more than 2 Z-scores from the reference are indicated in bold

In P1, the amount of osteoid was low; active osteoblasts and tetracycline double labels were seen only on cortical bone. Bone resorption was increased (Table 2), and there was peritrabecular fibrosis of bone marrow (Fig. 1). Dynamic parameters in cancellous bone could be measured in P2 and P3. In P2, dynamic parameters were within the normal range. In P3, no osteoclast was seen, and increased bone turnover rate (BFR/BV + 4.53 SD) associated with an increase in osteoid tissue was observed (Fig. 1). Double labels were seen in P3 ruling out the presence of pronounced osteomalacia. However, focal mineralization defects were identified as the presence of “osteoid islands” surrounded by mineralized bone (online resource).

### BMDD and OLS

**Fig. 2:**
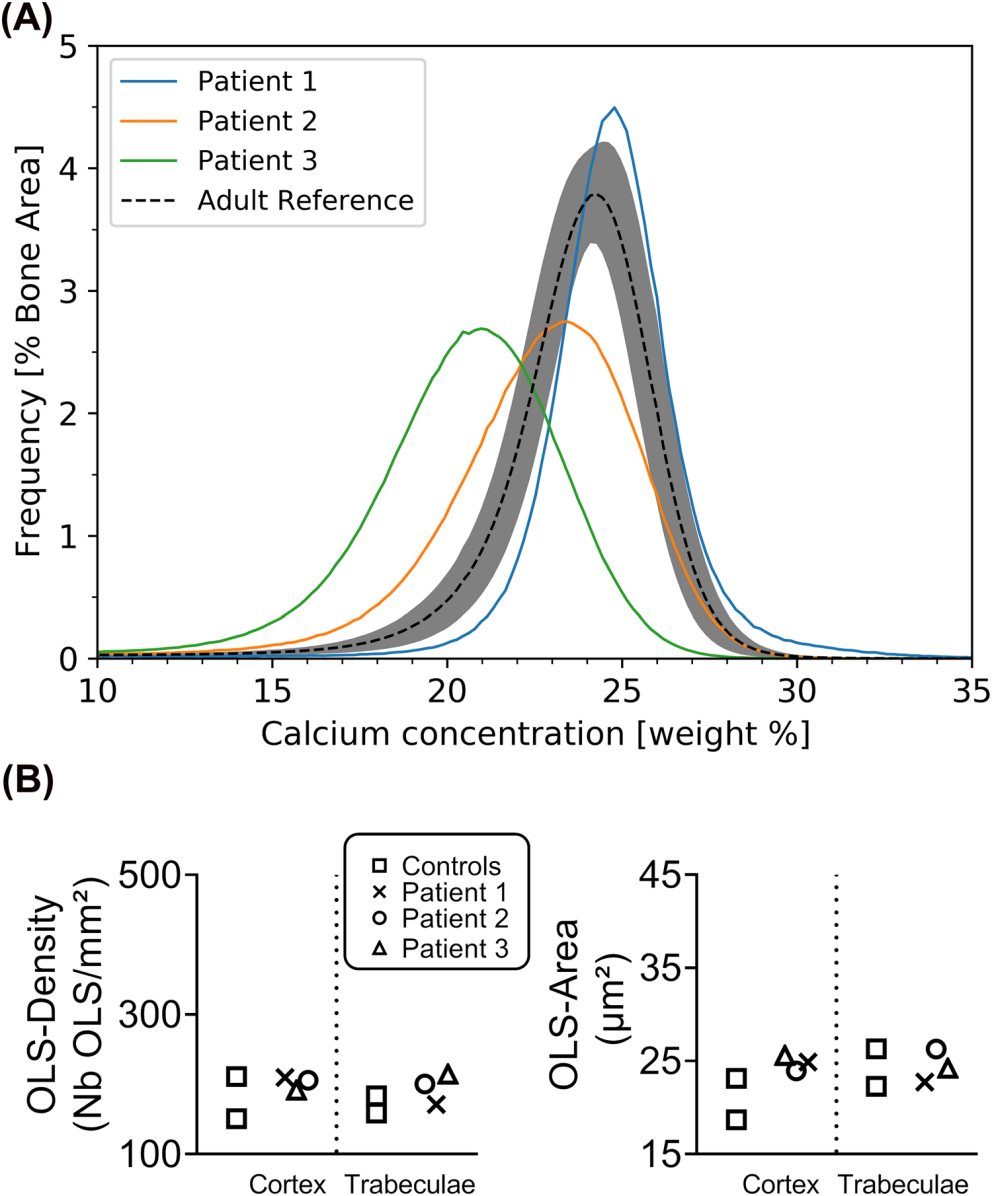
**A** shows the BMDD curves for trabecular bone of patients 1, 2, and 3, respectively. The black dashed line corresponds to the adult reference BMDD curve, the grey band depicts the corresponding standard deviation[10]. **B** show the results of the OLS analysis for OLS-density and OLS-area for cortical and trabecular compartments. Data are compared against results from 2 healthy women aged 36 and 42 years[12]

The three samples showed different bone matrix mineralization characteristics. Figure 2A shows the trabecular BMDD curves obtained for the three patients, as well as the adult reference curve for comparison [10]; online resource shows the resulting BMDD parameters for trabecular and cortical compartment. P1 showed a slight hypermineralization for the trabecular and cortical compartment as well as a decrease in the width of the BMDD (CaMeanTrab + 1.75 SD, CaWidthTrab − 2.04 SD, CaMeanCort + 1.59 SD, CaWidthCort − 1.53 SD) denoting a more homogenous mineralization compared to reference. In contrast, the trabecular BMDDs from P2 and P3 showed a considerable broadening of the curve. In P3, it was accompanied with a pronounced hypomineralization reflected in a large decrease in CaMean and CaPeak and a large increase in CaLow, respectively. The findings of qBEI were also reflected in the results from histomorphometry. The amount of osteoid volume and osteoid surface (Table 2) were consistent with the mineralization results, as a high turnover rate pointed towards a reduced degree of mineralization. Furthermore, similar with the histology pronounced focal mineralization defects could be spotted in P2 and P3 (online resource).

The OLS-density and OLS-area were similar to reference samples obtained from two healthy adults in both cortical and trabecular bone (Fig. 2B).

## Discussion

Our earlier study showed that recurrent fractures and spinal compression fractures were more prevalent in patients with APS1 than in their age- and gender-matched controls, although osteoporosis was rare [4]. This led us to investigate the bone histomorphometry and mineral properties in bone biopsies from three adults with APS1. Our findings indicated reduced trabecular thickness in all patients. Furthermore, the amount of osteoid was increased in P2 and P3, which together with hypomineralization measured by qBEI suggests a mineralization defect.

The reduced trabecular thickness was partly reversed by an increase in trabecular number. Therefore, bone volume was in the normal range for all patients. Increased trabecular number was not explained by any artifacts from the sampling procedure. Based on this cross-sectional study, we cannot define if the altered microstructure was formed by the modeling of growing skeleton or by remodeling during adulthood. The patients had multiple disease manifestations that could contribute to bone tissue abnormalities during their lifetime. Hypoparathyroidism is the most prevalent endocrinopathy in APS1 [13]. Deficiency of parathyroid hormone and low serum calcium levels lead to low bone turnover that has been associated with structural skeletal alterations, including higher cancellous bone volume and trabecular thickness [14, 15]. Cancellous BMDD is shifted to higher mineralization densities in patients with hypoparathyroidism [16]. In our study, P1 with longer duration of hypoparathyroidism (33 years) showed higher cancellous BMDD than P2 (5 years), but none showed higher cancellous bone volume or an increase in trabecular thickness. Lack of double strand labeling in cancellous bone in P1 resembles the significantly reduced cancellous bone formation in patients with hypoparathyroidism, whereas the increased eroded surface was in contrast to decreased resorptive activity reported previously [14].

Adrenal insufficiency and long-term glucocorticoid replacement may have had opposing effects on bone metabolism in comparison to hypoparathyroidism in P1 and P2. Their current hydrocortisone equivalent doses are near the recommended dose corresponding endogenous production of glucocorticoids [17]. Glucocorticoids act on osteoblasts, osteocytes, and osteoclasts as well as their precursors. Reduced bone formation at trabecular bone sites and increased endocortical resorption are the most consistent pathological findings of glucocorticoid excess [18]. Although meta-analysis showed that patients receiving glucocorticoid replacement due to adrenal insufficiency have a higher fracture risk [19], only modest data exist on the effects of glucocorticoid replacement on bone. Thus far no study has explored bone properties in the patients with adrenal insufficiency by bone histomorphometry.

In addition to lack of glucocorticoids and mineralocorticoids, adrenal insufficiency may lead to lack of adrenal androgens which act as precursors for peripheral conversion to more potent androgens and estrogens. P1 had used contraceptive pills for ovarian insufficiency and no DHEA supplementation. DHEA supplementation has been associated with higher areal BMD in female patients with adrenal insufficiency [20]. Placebo controlled trial with DHEA reversed ongoing loss of bone mineral density [21]. Androgens are important for the periosteal bone growth, whereas estrogens are crucial for the attainment and maintenance of trabecular bone mass in both females and males by affecting the differentiation and function of osteoblasts as well as osteoclasts [22–24]. Both our patients with hypogonadism showed decreased trabecular thickness and in female P1 increased eroded surface was evident. The early onset of hypogonadism may have decreased the accrual of peak bone mass in both patients. In male patients with hypogonadism, bone histomorphometry shows marked alterations in trabecular microarchitecture with decreased trabecular connectivity [25]. No study has explored the bone histomorphometry in patients with premature ovarian insufficiency.

In addition to endocrinopathies, altered kidney function and malabsorption may affect the mineral homeostasis. P3 had stable renal tubular acidosis since childhood. A previous study on bone histomorphometry in adult patients with longstanding renal tubular acidosis suggested that chronic metabolic acidosis may result in suppression of bone formation and resorption. BMD and bone formation rates were found to be lower, while osteoid volume and surface were increased [26]. Because of the multiple contributing factors, we were not able to determine whether renal tubular acidosis, hypogonadism, or exocrine pancreas insufficiency had had most impact on bone parameters in P3. All three patients had either exocrine pancreas insufficiency or intestinal dysfunction. Although all patients had adequate 25-hydroxy vitamin D levels, their intestinal absorption capacity of necessary nutrients may have been compromised during attainment of peak bone mass and afterwards.

The small number of participants and the lack of control subjects are the major limitations of the study. All three patients present with the severe phenotype of APS1, and the results may not apply to patients with milder disease severity.

In conclusion, we demonstrate variable and significant bone pathology on the tissue level in three patients with severe APS1 and osteoporosis. Altered mineralization and bone metabolism reflected some features of hormonal defects but the large number of endocrinopathies, often with opposing effects make it difficult to identify the main cause of observed pathologies and to identify potential primary skeletal effects. Osteoporosis treatment in patients with APS1 should consider the possibility of a mineralization defect.

## Supplementary Information

Below is the link to the electronic supplementary material.

Supplementary file1 (PDF 684 KB)

## Data Availability

Restrictions apply to the availability of data generated or analyzed during this study to preserve patient confidentiality. The corresponding author will on request detail the restrictions and any conditions under which access to some data may be provided.
