# Supplementary material for: Bone Tissue Evaluation Indicates Abnormal Mineralization in Patients with Autoimmune Polyendocrine Syndrome Type I: Report on Three Cases
Source: Calcif Tissue Int. 2023 Mar 22;112(6):675–82. doi: 10.1007/s00223-023-01077-0 (PMC10198912; doi:10.1007/s00223-023-01077-0)

Online resource for manuscript entitled "Bone tissue evaluation indicates abnormal mineralization in patients with autoimmune polyendocrine syndrome type I – three case reports" by Saila Laakso, Tong Xiaoyu, Stéphane Blouin, Petra Keplinger, Ville-Valtteri Välimäki, Heikki Kröger, Outi Mäkitie, Markus A. Hartmann, published in Calcified Tissue International.

Table 1. BMDD parameters obtained from the three patients in trabecular (Trab.) and cortical (Cort.) bone. Values are given with their corresponding Z-scores. Deviations more than 2 Z-scores from the reference are indicated in bold. Reference values are given as mean (SD)[10].

	**Patient 1**		**Patient 2**		**Patient 3**		**Reference**	
	Trab.	Cort.	Trab.	Cort.	Trab.	Cort.	Trab.	Cort.
CaMean [wt.% Ca]	24.29 (+1.75)	23.86 (+1.59)	22.24 (-1.76)	22.49 (-0.84)	19.96 (**-5.65**)	20.32 (**-4.68**)	23.26 (0.58)	22.96 (0.57)
CaPeak [wt.% Ca]	24.61 (+0.91)	24.35 (+0.85)	23.22 (-1.81)	23.66 (-0.44)	20.97 (**-6.23**)	21.32 (**-4.76**)	24.14 (0.51)	23.89 (0.54)
CaWidth [Δwt.% Ca]	3.29 (**-2.04**)	3.47 (-1.53)	5.72 (**+6.57**)	4.85 (+1.85)	5.89 (**+7.18**)	5.63 (**+3.76**)	3.87 (0.28)	4.09 (0.41)
CaLow [% Bone area]	3.25 (-1.04)	3.09 (-1.77)	8.66 (**+2.22**)	8.15 (+1.77)	21.22 (**+9.81**)	19.07 (**+9.38**)	4.98 (1.66)	5.62 (1.43)
CaHigh [% Bone area]	10.99 (**+2.21**)	6.74 (+1.25)	3.90 (-0.28)	3.72 (-0.05)	0.33 (-1.54)	0.61 (-1.38)	4.70 (2.84)	3.83 (2.33)

Figure 1. The upper row shows qBE images of the bone biopsy samples from the three patients with APS1. Bright pixels correspond to a high calcium content, darker pixels to a low calcium content. In each sample a cortical and trabecular compartment can be distinguished that are evaluated separately. In the qBE images of patients 2 and 3 areas with pronounced mineralization defects could be detected (a-c). The location of three of these areas are indicated with white dotted boxes. In the lower row, these areas are shown in larger magnification, and the mineralization defects can be found in the dotted boxes.

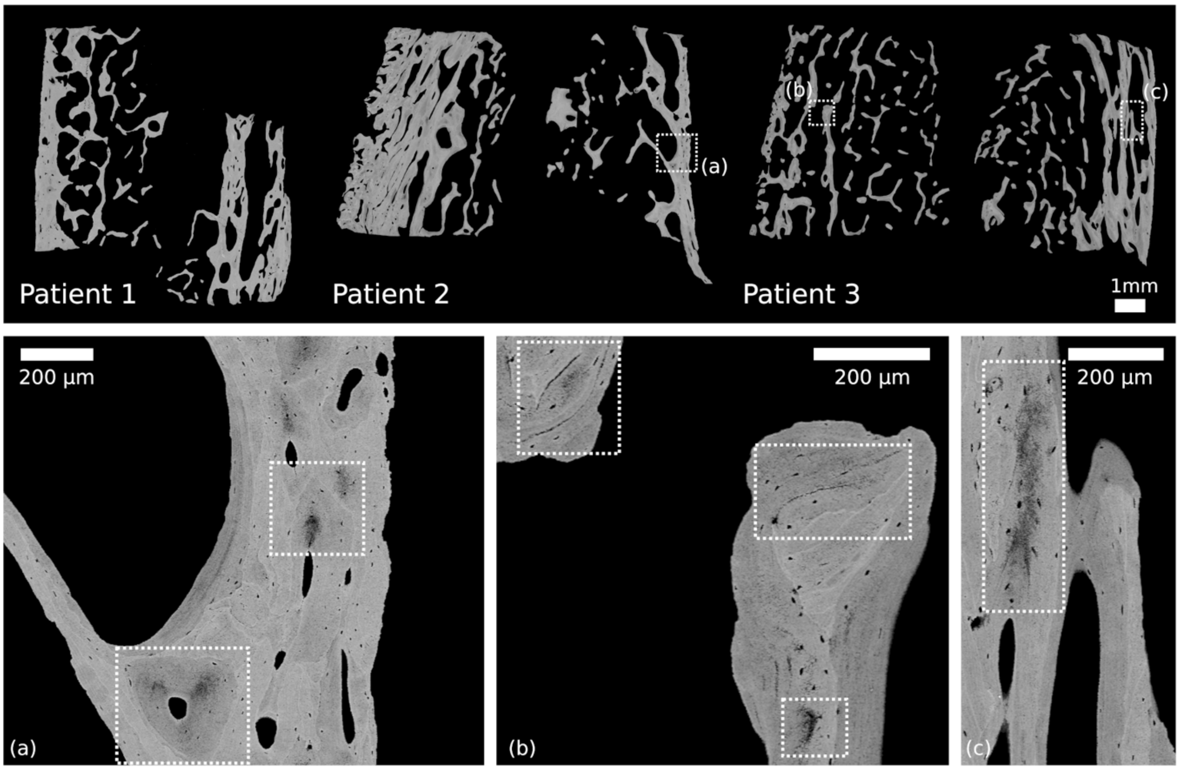

Supplement: Supplementary file 1 — Supplementary file1 (PDF 684 KB) [file 223_2023_1077_MOESM1_ESM.pdf]
